# Unveiling the Nutritional Quality of the Sicilian Strawberry Tree (*Arbutus unedo* L.), a Neglected Fruit Species

**DOI:** 10.3390/foods14152734

**Published:** 2025-08-05

**Authors:** Federica Litrenta, Vincenzo Nava, Ambrogina Albergamo, Angela Giorgia Potortì, Roberto Sturniolo, Vincenzo Lo Turco, Giuseppa Di Bella

**Affiliations:** Department of Biomedical, Dental, Morphological and Functional Images Sciences (BIOMORF), University of Messina, Viale Annunziata, 98100 Messina, Italy; felitrenta@unime.it (F.L.); vnava@unime.it (V.N.); agpotorti@unime.it (A.G.P.); roberto.sturniolo@studenti.unime.it (R.S.); vloturco@unime.it (V.L.T.); gdibella@unime.it (G.D.B.)

**Keywords:** *A. unedo*, Sicilian strawberry tree, chemical characterization, macronutrients, bioactive compounds, minerals

## Abstract

Although the strawberry tree (*A. unedo* L.) has been long considered a neglected species of the Mediterranean maquis, the valorization of its fruit production may enhance its socioeconomic value, especially in rural areas. In this study, strawberry trees from different Sicilian sites were investigated in terms of macronutrients, fatty acid (FA) composition, tocopherols, total phenols, carotenoids, and minerals. Sicilian berries were a good source of carbohydrates (mainly fructose, glucose and sucrose) and dietary fiber. They were low in fat; however, the FA composition revealed the abundance of unsaturated FAs over saturated FAs and an advantageous n-6/n-3 ratio. Additionally, Sicilian berries showed an inversed linoleic/α-linolenic acid ratio with respect to berries from other Mediterranean regions, that had previously investigated in literature. This evidence suggests that this ratio may have a chemotaxonomic relevance. Considering antioxidants, the fruits had levels of tocopherols, particularly α-tocopherol, total phenols and carotenoids similar to those of certain commercial fruits. Precious amounts of minerals, such as Ca, K, Zn and Fe were also determined. Interestingly, berries harvested near a Sicilian volcanic area had higher levels of minerals, as well as tocopherols, phenols and carotenoids, than fruits from other Sicilian sites, thereby advancing the hypothesis that fruits from volcanic areas may have a superior nutritional value. Overall, data from this study elaborated by a proper statistical analysis revealed that the geographical origin was a relevant variable to consider in the reliable study of this fruit species.

## 1. Introduction

*Arbutus unedo* L., also known as strawberry tree, is an evergreen fruit shrub with a circum-Mediterranean distribution. Specifically, the species occupies mainly the coastal area of all the northern African and southern European countries facing the Mediterranean Sea, where it grows spontaneously in drained and dry soils and rocky areas [1]. Similarly to other Mediterranean plants [2,3], *A. unedo* has long been referred as a neglected or underutilized species mainly because of its actual relatively limited growth area and small-scale use [4]. However, it is characterized by a multipurpose character, which may promote its sustainable deployment, thus enhancing its economic potential, especially in rural areas [5].

The strawberry tree is resilient to abiotic and biotic stressors, and able to regenerate quickly after fire, the latter characteristic being particularly important for forestation programs in countries, such as Greece, Italy, Portugal and Spain, where fires are common during the dry and hot summer. The species contributes also to ameliorate agroforestry areas by mitigating soil erosion and augmenting soil organic matter and to preserve the Mediterranean biodiversity by forming different plant associations and providing shelter and food for fungi, insects, birds etc. Additionally, the evergreen foliage, appealing flowers and fruits make the species an attractive choice for landscaping [5]. Other than that, *A. unedo* is well-known to produce fleshy berries that reach the optimum ripeness, determined by an intense red external colour, between November and December. Fruits are typically consumed fresh or, in some countries, used to produce food products, such as jam, jelly, vinegar, and alcoholic beverages, due to an intense and pleasant, sweet taste and a favourable chemical composition that mainly boosts a high sugar content -and hence energy value-, a high fibre content, and antioxidant polyphenols [6,7,8]. Because of the many recognized biological functions [9,10,11], the berries may find application as pharmaceuticals, dietary supplements and cosmetics [12,13,14,15,16]. Additionally, the high content of phenolics and dietary fibre could be considered as interesting ingredients for the expansion and development of new functional products [17,18]. However, the full exploitation of the strawberry tree is currently not so widespread, since it is first not recognized. This may be due not only to the absence of extensive and profitable cultivations, in turn heavily influenced by the lack of varietal selection and breeding programmes [19], but also to the scarcity of studies aiming to valorise the nutritional value of these fruits and enhance their multifaceted use.

A literature review over the past 15 years pointed out that only a few works addressed the chemical composition of *A. unedo* fruits in relation to study variables such as geographical origin, harvesting season and ripening process. These studies considered berries from different Mediterranean countries, mainly Turkey [20,21], Morocco [22,23,24,25], Tunisia [26], Algeria [27,28], Spain [29,30], Croatia [31,32], Portugal [33,34], and Italy [16,35,36].

**Figure 1:**
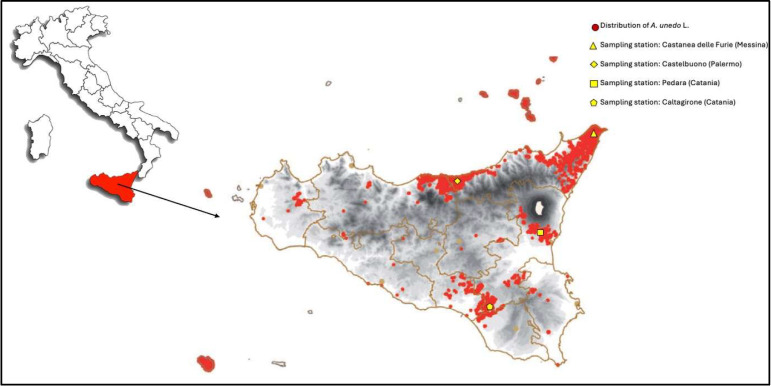
Map of Sicily showing the natural distribution of *A. unedo* L. and the four sampling stations of the study, which were purposely selected to fall within strawberry tree hotspots.

With respect to Italy, *A. unedo* grows wild almost along the entire coastal strip of the peninsula, and in the major and minor islands. The island of Sicily, particularly, enjoys a good spread of the strawberry tree in all those territories consisting of silicate substrates, namely northeastern Sicily and all the minor islands of volcanic origin, the southern slope of Etna, the coastal and subcoastal reliefs of north-central Sicily, and the southern Erei Mountains and adjacent reliefs (Figure 1), and it is typically in association with other species of the Mediterranean maquis, such as the mastic tree (*Pistacia lentiscus*) and the tree heather (*Erica arborea*). In this region, strawberry trees have stepped out from a small local production to a specialty product that receives appreciable consideration especially during the holiday season. Recent research available on the chemical composition of Italian and, particularly, Sicilian fruits is very limited and fragmented, since it focused only on single polyphenols and protein profile [16,35,36].

Hence, this study aims to expand knowledge about the overall nutritional quality of the Sicilian *A. unedo* fruits, by deepening macro-, micronutrients and bioactive compounds, with the main purpose to valorize local productions, stimulate the sustainable cultivation of the species and contribute to the development of (functional) food based on the strawberry tree as well.

## 2. Materials and Methods

### 2.1. Samples

*A. unedo* fruits were hand-harvested at their optimal ripening stage during November 2024 from wild specimens in four Sicilian hotspots of strawberry trees, namely Castanea delle Furie (province of Messina) and Castelbuono (province of Palermo)—which are located in the northeastern Sicily- and Caltagirone and Pedara (province of Catania)—which are placed in the southeastern area of the island. The taxonomic identification of the species was performed by the botanists of the University of Messina (Messina, Italy), where voucher specimens were also deposited.

Triplicate samples of ~1 kg of fresh strawberry trees each, were produced for each sampling station, for a total of 12 samples. Hence, every sample was placed in labelled plastic bags and transported to the laboratory on the same day of collection, where it was immediately frozen and stored at −40 °C. Detailed information on strawberry tree samples is provided in Table 1. For subsequent analysis, the fruits were always considered in their entirety, homogenized with a blender and analysed on a fresh weight (fw) basis.

### 2.2. Materials and Reagents

For the determination of crude fiber, protein and ash, the Megazyme assay kit and the Kjeldahl catalyst were provided respectively by International Ireland Ltd. (Wicklow, Ireland) and Carlo Erba (Milan, Italy). Organic solvents, such as n-hexane, n-heptane and acetone (reagent grade), ethyl acetate, acetonitrile and methanol (HPLC grade) were from J.T. Baker (Phillipsburg, NJ, USA). Ultrapure water (<5 mg/L TOC) was provided by a water purification system (BarnsteadSmart2Pure 12, Thermo Scientific, Milan, Italy). H_2_O_2_ (30%, *v*/*v*) and HNO_3_ (65%, *v*/*v*) (trace metal analysis grade) and ultrapure water (resistivity of 10 mΩ cm) were from Mallinckrodt Baker (Milan, Italy). The Folin–Ciocalteu reagent was obtained from Sigma-Aldrich (Steinheim, Germany). Reference standards of fatty acids methyl esters (FAMEs, C4–C24), single tocopherols (i.e., α-tocopherol, β-tocopherol, γ-tocopherol, and δ-tocopherol, 98% purity each), sugars (i.e., glucose, fructose, xylose, arabinose, sucrose, and maltose, ≥99% purity each), β-carotene (≥95% purity), gallic acid (≥99% purity) were purchased from Supelco (Bellefonte, PA, USA). Stock solutions of commercial of Re (internal standard), and other inorganic elements, such as Na, Ca, Mg, K, P, Fe, Co, Cu, Mn, Zn, Ni, Cr, Al, As, Cd, and Pb (1000 mg/L in 2% HNO_3_, each), were supplied Fluka (Milan, Italy).

### 2.3. Proximate Composition

The AOAC official protocols of analysis allowed to determine the proximate composition of strawberry tree fruits [37]. Crude fiber was measured according to the AOAC method 991.43. Specifically, duplicate samples (1.0 g each) were treated in parallel by a sequential heat stable enzymatic digestion (α-amylase, protease and amyloglucosidase) to remove starch and protein. Then, both digestates were treated with ethanol to precipitate fiber. The residues were filtered, washed with ethanol and acetone, dried and the mean weight was calculated. Moreover, at this stage, a residue was incubated at 500 °C until constant weight for ash analysis, while the other residue was tested for protein according to the AOAC method 976.05. In this respect, the residue underwent digestion with H_2_SO_4_, CuSeO_3_·2H_2_O, and K_2_SO_4_ by the SpeedDigester K-439 (Büchi, Flawil, Switzerland) and, subsequently, analyzed by the KjelMaster System K-375 provided with a steam generator, titrator, and pumps (Büchi, Switzerland). The digestate was treated with NaOH for generating NH_3_, that was steam distilled and titrated with HCl. Finally, the calculation of the protein (%) occurred by multiplying the nitrogen (%) by a conversion factor of 6.5. The crude fiber content was therefore equal to the mean weight of the dried residue minus the content of protein and ash. Total lipids were assessed by the AOAC method 920.39. A sample aliquot (15.0 g) underwent a Soxhlet extraction with n-heptane for 6 h. The lipid extract was evaporated to complete dryness by a rotating evaporator (Heidolph Instruments GmbH & Co., Schwabach, Germany), and the extraction yield was gravimetrically measured. Moisture was determined according to AOAC method 925.09 by oven drying the sample at 110 °C for 4 h and then recording the loss of sample weight. The phenol–sulphuric acid method was with slight modifications [38,39] was employed for the colorimetric determination of total carbohydrates. Around 100 mg of every sample were put in a test tube, mixed with 5 mL of 1.3 M HCl, and incubated in a water bath at 100 °C for 1 h. The tube was vortexed every half hour during the hydrolysis, and, then it was put into an ice-bath. After cooling, it was neutralized by adding 5 mL of 1.3 M NaOH, made up to 100 mL by distilled water, and filtered. Subsequently, 2 mL of the hydrolyzed sample was mixed with 1 mL of 5% aqueous solution of phenol and 5 mL of concentrated H_2_SO_4_. After allowing the mixture to stand for 10 min, it was vortexed for 30 s and placed for 20 min in a water bath at 30 °C for color development. Then, the absorbance at 490 nm was recorded by an UV spectrophotometer (UV-2401 PC, Shimadzu, Milan, Italy). Reference solutions of glucose were prepared in distilled water and treated in the same way as described for fruit samples to build up calibration curves useful for quantitative purposes. For every parameter of the proximate composition, all fruit samples were always analyzed in triplicate.

### 2.4. Sugars

Strawberry trees were screened for soluble sugars according to the method already described by Albergamo et al. [40]. Specifically, 10 g of every sample were mixed with 100 mL of acetonitrile/water (60:40, *v*/*v*), stirred in an ultrasound bath (30 min) and, subsequently, a magnetic stirrer (3 h). The supernatant was filtered, passed through a C18 Sep-Pak cartridge (Waters, Milford, MA, USA), and, analyzed by HPLC (Shimadzu LC10A System, Shimadzu, Kyoto, Japan) combined with a refractive index detector (Shimadzu RID-6A, Shimadzu, Kyoto, Japan). The chromatographic analysis occurred on a reverse phase propyl-amine-based column (Supelcosil LC-NH2, 25 cm × 4.6 mm, 5 μm ID, Supelco, Bellefonte, PA, USA) and under isocratic conditions with a mobile phase of acetonitrile/water (50:50) at a flow rate of 1 mL/min. The injection volume and the temperature of the column oven were respectively 10 µL and 25 °C. All samples were analyzed in triplicate along with analytical blanks. The LabSolutions software v. 5.53 (Shimadzu, Kyoto, Japan) aided the processing of chromatograms. The identification and quantification of individual sugars was performed with the help of commercial standards previously analyzed for their retention times and used for the construction of external calibration curves.

### 2.5. Fatty Acid (FA) Profile

The FA profile of strawberry trees was explored according to a protocol of sample preparation and analysis already reported in Di Bella et al. [41]. Every lipid extract was recovered through 1 mL of n-hexane, and trans-methylated with a methanol/sulfuric acid solution (9:1, *v*/*v*) at 80 °C for 10 min. The hydrocarbon layer was decanted and analyzed into a Master GC-DANI system (Dani Instrument, Milan, Italy), equipped with a split/splitless injector, and a flame ionization detector (FID). A Supelco SLB-IL100 capillary column (60 m × 0.25 mm, film thickness 0.20 µm) was employed for the chromatographic analysis. The oven temperature program was 130–210 °C (10 min holding) at 2 °C/min. Injector and FID temperatures were respectively set at 220 and 240 °C. Carrier gas was He, at a constant linear velocity of 30.0 cm/s. FID conditions were set as follows: sampling frequency: 25 Hz; gases: He (makeup), 25 mL/min, H_2_, 40 mL/min and air, 280 mL/min. Data were processed through the Clarity Chromatography Software v. 4.0.2 (Dani Instrument, Milan, Italy). FAMEs of nutritional interest were identified in strawberry tree samples with the help of the commercial standard mixture. The individual percentages of FAMEs were determined in relation to the total chromatogram area. Every sample was run in triplicate along with analytical blanks.

### 2.6. Tocopherols

In this study, α-, β-, γ-, and δ-tocopherols were determined in strawberry trees by following the protocol described by Lo Turco et al. with slight modifications [42]. Briefly, the lipid extract (0.1 g) of every fruit sample was resuspended with 1 mL of n-hexane and added with 1 mL of a methanolic KOH solution. The mixture was stirred, centrifuged and the n-hexane layer was collected and analyzed by high-performance liquid chromatography system coupled to a fluorescence detector (HPLC-FD, Prominence HPLC System with RF-20A Detector, Shimadzu, Milan, Italy). A LiChrosorb Si60 column (250 mm × 4.6 mm, ID 5 μm particle size) protected by a LiChroCART 4-4 guard column with the same stationary phase (Merck, Darmstadt, Germany) was introduced in the column oven at 40 °C. An isocratic chromatographic run was performed with n-hexane and ethyl acetate (90:10, *v*/*v*), and a flow rate of 0.8 mL/min. The LabSolutions software (ver. 5.10.153, Shimadzu, Kyoto, Japan) was used for instrument control and data processing. Tocopherol isomers were identified using detector excitation and emission wavelengths of 295 and 330 nm and quantified by an external calibration procedure. Every sample was investigated with triplicate measurements, along with suitable analytical blanks.

### 2.7. Total Carotenoids and Total Polyphenols

The extraction of total carotenoids was carried out according to the method employed by Ruiz-Rodríguez et al. [43], with slight modifications. In detail, 1 g of every sample was mixed with 50 mL of a solution of hexane: acetone (50:50, *v*/*v*), stirred for 30 min, and then, added with 5 mL of ultrapure water. The upper layer was collected, filtered and an aliquot (1 mL) was evaporated to dryness by a rotatory evaporator (Büchi Labortechnik AG, Flawil, Switzerland). The residue was reconstituted in 1 to 10 mL of acetone, and the absorbance of solution was measured by an UV-VIS spectrophotometer (UV-2401 PC, Shimadzu, Milan, Italy) at 450 nm wavelength as reported elsewhere [44]. An external calibration curve allowed to determine the concentration of total carotenoids, which were expressed as mg β-carotene equivalents (BCE) per kg of fruits.

The total phenolic content was determined by following the protocol of Monteiro et al. [45] with slight modifications. Briefly, an aliquot of every fruit sample (1 g) was mixed with 5 mL of a methanol:water solution (80:20, *v*/*v*). The mixture was sonicated for 1 h and centrifuged at 7000 rpm for 20 min (4 °C). The supernatant was collected, and the extraction was repeated by adding a further 5 mL of the solution to the pellet. The supernatants were combined, filtered and diluted 1:50 with distilled water. Hence, 0.2 mL of the extract were mixed with 10 mL of Folin Ciocalteau’s reagent and 8 mL of 7.5% Na_2_CO_3_ and incubated for two and a half hours in the dark. The absorbance readings were taken at 765 nm by using the UV-VIS spectrophotometer mentioned above. The total phenol content was calculated by an external standard curve and expressed as mg gallic acid equivalents (GAE) per kg of fruits. The total carotenoid and phenol content of all samples was always determined in triplicate, along with analytical blanks useful for subtracting the background noise of the method.

### 2.8. Inorganic Elements

Sample preparation and ICP-MS analysis were carried out according to the protocol reported by Lo Turco et al. [46]. Briefly, an 0.5 g of every fruit sample was mixed with 2 mL of H_2_O_2_, 8 mL of HNO_3_ and 1 mL of the internal standard Re (0.5 mg/L). The digestion process occurred in a microwave digestion system (Ethos 1, Milestone, Bergamo, Italy) with a temperature program consisting of 0–200 °C in 10 min, and 200 °C held for 15 min, and a constant microwave power of 1000 W. Then, the digested samples were cooled to room temperature and suitably diluted with ultrapure water. A quadrupole ICP-MS (iCAP Q, Thermo Scientific, Waltham, MA, USA) was employed for the analytical determinations, that were performed according to the operating conditions reported in Table 2. For quantification, external six-point calibration curves were built up for each analyte with the help of multielemental standard solutions. Data acquisition and handling were conducted using the Qtegra™ Intelligent Scientific Data System software (Thermo Scientific, Waltham, MA, USA). All fruit samples were analyzed three times, along with analytical blanks to check for cross contamination.

### 2.9. Statistical Analysis

All statistical interpretations were performed using the SPSS 13.0 software for Windows (SPSS Inc., Chicago, IL, USA). The statistical analysis was conducted with a data matrix consisting of 12 cases (i.e., 12 fruit samples) and 48 variables (i.e., all nutritional characteristics measured in this study), and the cases were divided into four groups according to geographical origin of strawberry trees (i.e., the four sampling sites). In the dataset, δ-tocopherol and maltose were not included because they were always below the limit of detection (LOD). When the concentrations were below the LOQ as in the case of Pb, which was below the LOQ in 50% of the samples, they were replaced by half of the limit of detection (LOD/2). On this matrix, a one-way ANOVA with Kruskal-Wallis post-hoc multiple comparison test was first run to assess the significance of differences among strawberry trees according to the sampling area. Statical significance was set at *p* < 0.05. Next, the data set was normalized to obtain the independence of the scaling factors of different variables. After checking the adequacy of the initial data using the Kaiser-Meyer-Olkin (KMO) test and Bartlett’s test, the data were subjected to principal component analysis (PCA) to differentiate the fruit samples of different geographical origin.

## 3. Results and Discussion

### 3.1. Proximate Composition

The proximate composition of the Sicilian strawberry tree is reported in Table 3. Overall, the fruit was characterized by a consistent moisture (range: 46.82–56.31%) responsible for its valuable fleshyness and juiciness, and, to follow, appreciable levels of carbohydrates (range: 18.00–24.62%) and dietary fiber (range: 9.81–13.00%). Even taking into account the natural variation, the strawberry tree may be defined as a “natural source of fiber” due to a content of dietary fiber far greater than 3%, according to the Regulation (EC) 1924/2006 [47]. On the other hand, protein, lipids and ash were much lower, ranging respectively between 1.63–2.50%, 1.65–2.43%, and 1.13–2.58%.

The comparison of berries from different sites revealed that a similar proximate composition was generally observed between samples from northeastern Sicily (i.e., samples S1 and S2), as well as between samples from the southeastern island (i.e., samples S3 and S4). In fact, samples S1 and S2 were characterized by a significantly higher moisture content than samples S3 and S4 (56.27–56.31% vs. 46.82–47.03%, *p* < 0.05) and, consequently, a significantly lower content of carbohydrates (18.00–19.87% vs. 22.53–24.62%, *p* < 0.05), protein (1.63–1.68% vs. 2.48–2.50%, *p* < 0.05), lipids (1.65–1.75% vs. 2.34–2.43%, *p* < 0.05), and ash (1.13–1.28% vs. 2.00–2.58%, *p* < 0.05). However, dietary fibre did not differ significantly between the four sampling sites (9.81–9.88% vs. 12.84–13.00%, *p* > 0.05) (Table 3). Overall, these results confirm that the geopedoclimatic context remarkably affects the metabolism of *A. unedo* and, hence, the nutritional quality of its fruits.

Literature of the last 15 years reported not comparable profile of macronutrients of strawberry trees, while confirming that carbohydrates and fiber are the most abundant costituents. Ruiz-Rodríguez et al. [43] focused on fresh and ripe fruits collected during 2007–2009 from two Spanish sites, namely Madrid (center of Spain) and Cáceres (west of Spain). They highlighted that not only the site of origin, but also the sampling season affected the content of moisture (range: 46.82–71.89%), carbohydrates (range: 14.11–31.55%), dietary fiber (range: 10.04–22.27%), protein (range: 0.58–1.19%), and lipids (range: 0.30–0.78%) of strawberry trees, thus confirming the variability of the proximate composition. Vidrih et al. [48] reported that fresh and ripe strawberry trees from Vrsar, a seaside village of Istria County, had a moisture content of 46.6%, while protein, lipids, crude fiber and ash amounted respectively to 0.82%, 0.43%, 18.49% and 0.48%. However, the sample size was too small to make any assumptions about the compositional variability of the fruits. Algerian fruits collected from different sites on Tell Atlas Mountains during 2012 and 2013 showed a very high moisture content (63.7–74.13%) followed by carbohydrates (12.68–18.93%) and crude fiber (9.28–14.13%), and minor amounts of protein (2.26–3.55%), ash (0.53–0.88%) and lipids (0.51–0.89%) [27]. In the same study, Boussalah et al. highlighted that the harvesting season had more influence than location on the nutritional composition of fruits [27].

### 3.2. Sugars

The profile of soluble sugars of Sicilian *A. unedo* fruits is reported in Table 4. Fructose and glucose represented the most abundant monosaccharides in all samples with a range of 6.37–9.37 g/100 g and 2.24–5.40 g/100 g, respectively, and, thus, amounting to ~48–60% of total carbohydrates illustrated in Table 3. In the same profile, sucrose (range: 0.96–1.16 g/100 g), xylose (range: 0.25–0.85 g/100 g), and arabinose (range: 0.07–0.22 g/100 g) were also present in lower amounts; while maltose was lower than its limit of detection (<LOD, <0.001 g/100 g) in all samples. This study confirmed that *A. unedo* berries have an unusual profile of soluble sugars compared to other fruit species, particularly with regard to glucose, fructose and sucrose. In fact, experimental data not only show that the glucose/fructose ratio, which is indicative of the fruit’s invertase activity, was lower (0.27–0.57) than that of other common fruits (e.g., grape, strawberry, and blueberry), which have a glucose/fructose ratio ≥ 1 [49,50], but also that, unexpectedly, low amounts of sucrose were detected. During fruit ripening, invertase catalyzes the hydrolysis of sucrose to form glucose and fructose, the amounts of which should be the same and, thus, in a ratio near to 1. Moreover, the activity of invertase is inversely related to the accumulation of sucrose, meaning that a low enzymatic activity typically leads to an increased sucrose content [51]. In this instance, however, the assumed low invertase activity of strawberry trees was accompanied by a low sucrose content. Hence, not only the hydrolysis of sucrose but also other metabolic pathways may be involved in shaping the profile of soluble sugars of *A. unedo* berries [43]. Moreover, the higher levels of fructose, and the lower contents of glucose and sucrose of this profile could be responsible for the intense pleasant, and sweet taste of ripe strawberry trees, since fructose is the sweetest naturally occurring carbohydrate [43]. Interestingly, the little amounts of xylose and arabinose revealed in the sugar profile of strawberry trees may represent cell wall sugars [34].

When considering the geographical origin of samples, statistically significant variations of soluble sugars were observed in strawberry trees. In fact, apart from sucrose, which was found to be not significantly different in all samples (*p* > 0.05), the other monosaccharides were significantly higher (*p* < 0.05) in samples from the southeastern Sicily than fruits from the northeastern area. Specifically, fructose varied from 6.37–6.93 g/100 g (*p* > 0.05) in samples S1 and S2 to 8.60–9.37 g/100 g (*p* > 0.05) in fruits S3 and S4. Similarly, berries from S3 and S4 sites had greater amounts of glucose than fruits from S1 and S2 (i.e., 4.55–5.40 g/100 g vs. 1.91–2.24 g/100 g, *p* < 0.05). The same trend was observed also for xylose and arabinose (Table 4).

A literature review pointed out that ripe and fresh A. unedo fruits were investigated for main soluble sugars, such as glucose, fructose and sucrose, in many previous works by exploring different variables. However, regardless of the study variables (e.g., geographical origin, ripening stage…), comparable profiles were generally obtained, which resulted in findings that are in line with those of this study. For example, strawberry trees from different Turkish provinces confirmed to have high levels of fructose (7–11 g/100 g), followed by glucose (3.91–6.10 g/100 g) and small amounts of sucrose (0.26–1.56 g/100 g) [20,21]. In Algerian berries collected from four sampling areas on the Tell Atlas Mountains, fructose and glucose amounted to 5.52–8.44 g/100 g and 2.90–5.24 g/100 g, respectively [27]. Croatian strawberry trees showed a comparable content of glucose (5.27 g/100 g) but a slightly higher content of fructose than our study (16.62 g/100 g) [48]. Similarly, ripe berries gathered during different harvesting seasons (2007, 2008 and 2009) and from two Spanish areas with different environmental conditions, had glucose and fructose ranging between 2.34–6.50 g/100 g and 3.65–13 g/100 g, respectively [43].

### 3.3. Fatty Acid (FA) Profile

The FA profile of Sicilian strawberry trees is shown in Table 5. Overall, the berry is characterized by the highest levels of polyunsaturated fatty acids (PUFA, 49.33–60.57%), followed by saturated fatty acids (SFA, 22.54–30.93%) and monounsaturated fatty acids (MUFA, 10.65–21.80%). Palmitic (C16:0) and stearic (C18:0) acids were the predominant SFAs (i.e., 15.43–21.66% and 5.01–9.34%, respectively), while MUFAs were made up almost entirely of oleic acid (C18:1 n-9), with concentrations ranging from 7.37% to 19.20%. Linoleic (C18:2 n-6) and α-linolenic (C18:3 n-6) acids were the most represented PUFAs (i.e., 30.58–37.35%) (Table 5).

As already discussed in Section 3.1, the lipid content of strawberry trees is very low (1.65–2.43%), mainly due to their high moisture content, therefore, confirming that fruits are generally considered as a low-fat food. However, the study of the FA profile is relevant, since FAs with different chain length and unsaturation degree have notoriously a differential effect on human health, from energy production and storage, construction of cell membranes, to gene expression and hormone production [52]. Hence, certain indices have been calculated in this study to assess the nutritional value of the Sicilian strawberry tree in terms of FA content. It is already well known that the higher the unsaturated/saturated fatty acid (UFA/SFA) ratio, the more precious the nutritional quality of food. In this study, this ratio varied between 2.23–3.43, thus, demonstrating the greater and profitable content of UFA than SFA in strawberry trees. On the other hand, the n-3/n-6 ratio was close to 1 (i.e., range of 1.61–1.63), thereby confirming that the consumption of these fruits supports the normal functioning of physiological processes and the reduction of cardiovascular risk. However, the favorable n-6/n-3 ratio of strawberry trees was already highlighted by Oliveira et al. [53].

Differences, sometimes statistically significant, were found in the FA composition of samples of diverse geographic origin (Table 5). SFA, for example, resulted significantly different in all samples, being at lower levels in S1 and S2 fruits (i.e., 24.71% and 22.54%, *p* < 0.05) than in S3 and S4 samples (i.e., 28.56% and 30.93%, *p* < 0.05). This trend was also observed for palmitic acid, the most abundant SFA, which was determined at lower amounts in strawberry trees from northeastern Sicily (i.e., sample S1: 16.54% and sample S2: 15.43%, *p* < 0.05) than in fruits from the southeastern island (i.e., sample S3: 21.66% and sample S4: 19.22%, *p* < 0.05). Conversely, the other predominant SFA, namely stearic acid, was found at the lowest levels in samples S2 and S3 (i.e., 5.48% and 5.01%, *p* > 0.05) and at the highest levels in S4 fruits (i.e., 9.34%, *p* < 0.05). The highest MUFA percentage was found in samples from northeastern Sicily (i.e., sample S1: 21.32% and sample S2: 21.80%, *p* > 0.05) and at significantly lower levels in fruits the southestern area (i.e., sample S3: 10.65% and sample S4: 19.50%, *p* < 0.05). This trend was comparable to that of the most abundant MUFA, namely oleic acid, which was determined at the highest content in strawberry trees from samples S1 and S2 (i.e., 18.27% and 19.20%, *p* < 0.05) and, to follow, in samples S3 and S4 (i.e., 7.37% and 15.86%, *p* < 0.05). However, the behavior of PUFA was different from that of MUFA, since strawberry trees from sample S3 (southeastern Sicily) showed the highest PUFA content (i.e., 60.57%), followed by samples collected in northeastern Sicily (i.e., sample S1: 54.06% and sample S2: 55.43%, *p* > 0.05). Consequently, PUFA were at the lowest levels in fruits from sample S4 (i.e., 49.33%, *p* < 0.05). Accordingly, the highest and lowest levels of predominant PUFAs, such as linoleic and α-linolenic acids, were determined respectively in fruits from sample S3 (i.e., respectively 37.35% and 23.22%, *p* < 0.05) and S4 (i.e., respectively 30.58% and 18.75%, *p* < 0.05) (Table 5).

In the last 15 years, only a few studies explored the FA composition of A. unedo fruits, and a high variability resulted from the comparison of this work with our findings. Oliveira et al. [53], for example, focused on unripe, intermediate and ripe strawberry trees from northeastern Portugal and found out that the ripening process leads to a slight decrease of SFA (i.e., from 12.64% to 10.04%) and MUFA (i.e., from 33.49% to 27.00%) and a concomitant increase of PUFA (i.e., from 52.47% to 62.01%). However, the Portuguese ripe fruits did not show a comparable FA composition to that of Sicilian strawberry trees. In fact, they had lower amounts of palmitic (i.e., 5.92%) and stearic (i.e., 3.78%) acids responsible for a lower SFA content with respect to the Sicilian counterpart. Conversely, MUFA and PUFA were more abundant than our study, due to higher percentages of oleic (26.75%) and α-linolenic (43.07%) acids. In another study, Morales et al. [54] elucidated the FA composition of Spanish *A. unedo* fruits. They highlighted that the ripe fruits had mean SFA (19.62%), and MUFA (24.90%), which were respectively less and more abundant than those observed the present study. Moreover, while the main FAs of these fractions were confirmed to be palmitic, stearic and oleic acids, they were not comparable to the FAs of Sicilian fruits (i.e., 10.54%, 4.34% and 24.82%, respectively). However, the PUFA content (55.48%) was more similar to that in our study, although the most abundant FA was α-linolenic acid (31.26%), followed by linoleic acid (24.22%). Algerian ripe fruits sampled from four sites on the Tell Atlas Mountains [27] were characterized by highly variable FA profiles, but they were generally more comparable to those described in previous research [53,54] than to those in the present study. In fact, SFA ranged from 10.83% to 17.60%, with the most abundant palmitic acid amounting to 7.22–11.44%, and MUFA varied from 17.09–30.50%, being oleic acid comprised between 16.53–25.69%. Finally, PUFA were between 56.34–68.18%, with the predominant α-linolenic acid making up between 30.53% and 45.10% of the total fraction. Based on the evidence of previous literature and this study, while strawberry trees from diverse Mediterranean regions were characterized by a higher contribution of α-linolenic acid than linoleic acid, Sicilian berries had linoleic acid as the most abundant PUFA, followed by α-linolenic acid. This, in turn, inevitably led to a reversed linoleic acid/α-linolenic acid ratio.

Intraspecific chemical variability is quite common in the plant kingdom. It depends on a combination of factors related to the environment and genetics, as well as the anatomical and physiological characteristics of plants. Keeping in mind the geopedoclimatic differences between the areas studied so far (i.e., Portugal, Spain and Algeria), a certain relationship between chemical variations and habitats may be suggested. In fact, one could put forward the hypothesis that the study of the FA profile, with a particular focus on the linoleic acid/α-linoleic acid ratio, may have a chemotaxonomic relevance and be a useful tool for population discrimination within the *A. unedo* species. However, additional analyses of environmental and ecological factors of the study area and eco-physiological traits of the plants are undoubtedly needed.

Although minor FA fluctuations may occurr under the influence of environment, recent evidence has already highlighted that the FA profile may have reliable utility for delimitations at different taxonomical and intraspecific levels in lipidomic based studies [55,56,57].

### 3.4. Tocopherols

Tocopherols are widely known as lipophilic antioxidants, which can scavenge radicals in lipid oxidation. Plants produce these compounds to varying degrees and for their own protection against oxidative damage. At the same time, ensure the intake of tocopherols through the proper intake of vegetables, nuts, fruits, and vegetable oils may provide diverse health-promoting effects to consumers.

Single and total tocopherols of Sicilian *A. unedo* berries are reported in Table 6. According to the obtained data, α-tocopherol was the most abundant isomer in fruits (range: 6.23–8.58 mg/kg), followed by γ-tocopherol (range: 2.05–3.20 mg/kg) and β-tocopherol (range: 0.08–0.35 mg/kg). The δ-isoform was lower than the limit of detection (LOD) in all samples. Overall, the *A. unedo* fruit demonstrated to be a good source of tocopherols. Although several variables cause the tocopherol profile to vary highly even within the same fruit species, α-tocopherol of strawberry tree results comparable to those found in other edible fruits, such as blueberries (5.8 mg/kg), peaches (7.1 mg/kg), red raspberries (8.5 mg/kg) and banana (5.2 mg/kg) [58,59].

Considering the geographical origin, strawberry trees from the northeastern Sicily had significantly different single and total tocopherols than samples from the southeastern area of the island. For example, samples S1 and S2 were characterized by similar total tocopherol contents (i.e., 8.55 mg/kg and 8.65 mg/kg, *p* > 0.05), which, however, were significantly lower (*p* < 0.05) than those determined in S3 and S4 fruits (i.e., 11.19 mg/kg and 11.73 mg/kg, *p* > 0.05). Accordingly, α-, β and γ-tocopherols showed the same statistical behavior between samples (Table 6).

A literature overview from the last 15 years pointed out that only a few works explored the tocopherol profile of strawberry trees. These studies confirmed that α- and γ-tocopherols were the most abundant isoforms, although not comparable data were produced. Hence, the geopedoclimatic context again demonstrated to influence the nutritional profile of strawberry trees, also in terms of micronutrients. Oliveira et al. [53] demonstrated that the ripening process of strawberry trees led to a sharp decrease of tocopherols, to the extent that, in ripe fruits, the δ-isomer became non-detectable, the β-isoform was present in trace amounts (i.e.,0.39 mg/kg), while α- and γ-tocopherols amounted respectively to 32.16 mg/kg and 25.09 mg/kg. On this basis, the absence of δ-tocopherol noticed in Sicilian strawberry trees could be related to the selection and analysis of ripe fruits. In another study, Portuguese *A. unedo* berries had a variable content of α- and γ-tocopherols (i.e., 10–40 mg/kg and 10–60 mg/kg, respectively), depending on the sampling site [33]. The Spanish strawberry tree had a much higher total tocopherol content (38.8 mg/kg) than the fruits investigated in this study, mainly due to a greater amount of α-tocopherol (34.9 mg/kg). However, γ-tocopherol was revealed at comparable levels (2.6 mg/kg) [54]. Interestingly, the authors of this study affirmed that the antioxidant α-tocopherol can contribute to the inhibition of the lipid peroxidation of the fruit itself.

### 3.5. Total Carotenoids and Total Polyphenols

Total carotenoids and polyphenols of the Sicilian strawberry tree are reported in Table 7. Overall, total carotenoids varied between 9.60–15.45 mg BCE/kg, while total polyphenols ranged between 3027.61–3666.17 mg GAE/100 g.

The comparative assessment of these micronutrients in other fruits is quite hard, due to the high variability of these bioactives within the same species related to many intrinsic (i.e., cultivar, genotype etc.) and extrinsic (i.e., geographical origin, processing, storage etc.) factors. However, well known berries, such as blueberries, black mulberries, barberries and gooseberries, were characterized by levels of total polyphenols similar to those of Sicilian strawberry trees (i.e., 2784 mg GAE/100 g, 2237 mg GAE/100 g and 3450 mg GAE/100 g, and 2611 mg GAE/100 g, respectively) [60]. Moreover, recent studies on nectarine reported total carotenoids in line with our study (range; 2.4–35.1 mg/kg) [61,62].

With respect to the geographical origin, samples from the southeastern area of the island (i.e., sample S3 and S4) showed higher contents of carotenoids and polyphenols with respect to fruits from the northeastern area (i.e., sample S1 and S2). However, the statistical significance could not always be established (Table 7).

Research over the past 15 years focused more on polyphenols than carotenoids in *A. unedo* fruits. By considering fresh and ripe berries, lower polyphenol contents were generally outlined. In fact, Ruiz-Rodríguez et al. analyzed strawberry trees from three different seasons (i.e., 2007–2009) and two Spanish localities (i.e., Madrid and Cáceres) and revealed total phenols varying between 951.7–19.736 mg/100 g [43]. The statistical analysis confirmed also that the harvesting year, and thus the changing climate conditions, has a stronger influence than the geographical location [43]. More recently, Spanish fruits harvested at different harvesting seasons and ripening stages were considered [30]. During all the harvesting seasons, the highest and the lowest phenol contents were determined in unripe (2671–3612 mg/100 g) and intermediate-ripening stage (1309–2018 mg/100 g) fruits, while ripe strawberry trees experienced a slight increase (2071–2206 mg/100 g). Moreover, the phenol contents positively correlated with the high antioxidant activity of the fruits [30]. The findings of Izcara et al. [30] agree with the ones already reported by Oliveira et al. [53] who showed slightly lower values in unripe berries (2535 mg/100 g) than ripe strawberry trees (2681 mg/100 g). Finally, three previous studies on ripe berries from different Croatian sites agreed in revealing even lower total phenol contents (general range: 59.95–850.02 mg/100 g) [31,48,63]. Many of these studies deepened the study of polyphenol profile by revealing that the strawberry tree contains several hydroxycinnamic and hydroxybenzoic acids, with gallic acid being the highest phenolic acid, flavonols (e.g., quercetin, quercetin-3-glucoside, rutin, and kaempferol), and flavanols, with gallocatechin and catechin being the most abundant compounds [22,23,30,32].

To the best knowledge of the authors, only Šic Žlabur et al. [31] spectrophotometrically determined total carotenoids in fresh and ripe *A. unedo* fruits from diverse Croatian localities. This previous research revealed a content ranging from 60 to 270 mg/kg, which is much higher than that found in the present study, and affirmed that β-carotene and lycopene were the major carotenes in these fruits [31]. However, a recent study explored in detail the carotenoid profile of Spanish *A. unedo* fruits and found out that violaxanthin and neoxanthin were the most abundant compounds, followed by lower amounts of antheraxanthin, lutein, zeaxanthin, β-cryptoxanthin and β-carotene. Moreover, most of these carotenoids were naturally esterified with saturated fatty acids [29].

### 3.6. Inorganic Elements

The profile of inorganic elements of Sicilian *A. unedo* berries is shown in Table 8.

Considering macroelements, they were revealed in fruits according to the quantitative order: K > Ca ≅ P > Na ≅ Mg, while the microelements followed the sequence: Fe ≅ Zn > Cu > Mn > Mo. On the other hand, toxic and potentially toxic elements were for the first time detected in strawberry trees in the order: Al > Cr > Ni ≅ Cd > As ≅ Pb (Table 8). The Regulation (EU) no. 2023/915 [64] has fixed maximum levels for several contaminants in foods, including heavy metals, and the maximum levels for Pb and Cd allowed in strawberry trees are equal to 0.030 and 0.20 mg/kg, respectively. On this basis, the Sicilian strawberry can be considered safe, since all samples had contents of Cd and Pb below the legislative limits.

The geographical origin affected the elemental profile of *A. unedo* berries, because of statistically significant differences found in most macro- and microelements of fruits from Castelbuono and Castanea delle Furie, on the one hand, and samples from Caltagirone (S1 and S2) and Pedara (S3 and S4), on the other hand (Table 8). Among macrominerals, the most abundant K and Ca were at significantly higher levels (*p* < 0.05) in samples S3 and S4 (i.e., 3425.87–3940.53 mg/kg and 1196.50–1286.03 mg/kg, respectively) than samples S1 and S2 (i.e., 2435.99–2517.74 mg/kg and 938.87–948.61 mg/kg, respectively). The same trend was observed in microelements such as Fe Cu and Mn, which were significantly more concentrated (*p* < 0.05) in samples S3 and S4 (i.e., 7.74–9.27 mg/kg, 1.47–2.64 mg/kg and 1.03–2.00 mg/kg, respectively) than fruits from sample S1 and S2 (i.e., 5.48–6.11 mg/kg, 0.83–0.88 mg/kg and 0.60–0.61 mg/kg, respectively). However, toxic and potentially toxic elements showed a variable behavior. In fact, Cr and Al were at significantly higher concentrations (*p* < 0.05) in samples S1 and S2 (i.e., 1.71–1.72 mg/kg and 4.14–4.16 mg/kg, respectively) than samples S3 and S4 (i.e., 0.75–0.76 mg/kg and 3.35–3.77 mg/kg, respectively), while Ni (range: 0.010–0.033 mg/kg, *p* > 0.05), Cd (range: 0.012–0.022 mg/kg, *p* > 0.05), Pb (range: <LOD-0.014 mg/kg, *p* >0.05), and As (range: 0.011–0.015 mg/kg, *p* > 0.05) were revealed at non significantly different amounts in all samples (Table 8).

The high concentrations of all macroelements (i.e., Na, Ca, Mg, K, and P) and most microelements (i.e., Fe, Cu, and Mn) in berries from Caltagirone and Pedara could be explained by the proximity of these sampling sites to the volcanic area of Mount Etna, exposing the surrounding vegetation to volcanic ash, which mainly consists of aluminum, iron and sulfur with alkali and alkaline earth metal oxides such as CaO, MgO, Na_2_O, and K_2_O.

Although large amounts of ash have notoriously the potential to alter the vegetation structure, and damage soil water and nutrient balance, small quantities can act as a beneficial fertilizer of essential elements, like Ca, Mg, Na, K, Cu, Fe, and Mn, which are necessary for many biological processes in plants. As a result, literature highlighted that crops grown in volcanic soils or in presence of volcanic ash experience not only a notable increase in inorganic elements, but also in the synthesis of secondary metabolites, especially polyphenols, thus, enhancing their antioxidant capacity [65,66,67]. These findings are consistent with the results of this study, since strawberry trees collected near Mount Etna (i.e., southeastern area of Sicily) would be richer in beneficial macro- and microelements, as well as antioxidants, such as polyphenols, carotenoids and tocopherols, as discussed in the previous paragraphs. Clearly, such a hypothesis must be validated by further and advanced analysis that, complementing the spectrophotometric assays, can investigate the profile of individual polyphenols and carotenoids in greater depth.

Interestingly, no significant heavy metal accumulation was detected in these fruit samples, thereby indicating that the benefits of volcanic ash can be harnessed without associated toxicity risks.

Recent literature showed that the element profile of fresh and ripe strawberry trees was barely addressed in a few studies, with contrasting results regarding the predominant elements discussed so far. The element profile of berries from diverse Algerian sites [27] and from Croatia [48] was comparable to that of the present study, and the slight variability of data could be explained by the different geographical origin of fruits. In fact, in these previous works, K and Ca respectively amounted to 1186.10–3486.0 mg/kg and 315.0–959.4 mg/kg, while microelements such as Fe and Zn ranged respectively between 7.01–17.02 mg/kg and 2.30–4.63 mg/kg [27,48].

On the other hand, berries from different harvesting seasons (2007, 2008 and 2009) and from Spanish sites with different pedoclimatic contexts were characterized by contents of macro- and microelements much higher, and, thus, not comparable to the mineral fingerprint of Sicilian strawberries. Indeed, K and Ca ranged between 79.72–323.14 g/100 g and 40.54–104.12 g/100 g, respectively; Fe and Zn varied between 0.354–18.56 g/100 g and 0.188–0.762 g/100 g [43].

### 3.7. Principal Component Analysis (PCA)

PCA was applied to the normalized data matrix by using as variables all those compounds found to be significantly different in berries according to the sampling site. Analysis of the correlation matrix shows that the highest positive correlations were observed for PUFA-C18:2n-6 (0.994), UFA/SFA-Zn (0.951), moisture-Pb (0.990), Pb-Cr (0.992), Zn-Cr (0.983) and Mn-Cu (0.977), whereas the highest negative correlations were observed for UFA/SFA-SFA (−0.998), proteins-Pb (−0.989), proteins-Cr (−0.985), Zn-glucose (−0.964), Pb-K (−0.963) and lipids-Cr (−0.947).

According to the Kaiser-Guttman criterion, three principal components (PCs) with eigenvalues greater than one (i.e., 28.444, 7.292 and 1.977) were extracted, which together explain 94.283% of the total variance (i.e., 71.110%, 18.230% and 4.943%, respectively). Since there were no variables with low saturation in each factor and the communality was always higher than 0.751, the extracted PCs were able to reproduce all the variables well. The first PC has the highest positive correlation with stearic acid (C18:0), total polyphenols and, to a lesser extent, with Mn, while negative correlations are observed for total carotenoids and C20:1n-9; the second PC shows positive correlations with MUFA, C18:1n-9, and, to a lesser extent, Al, while it shows negative correlations with PUFA, linoleic (C18:2n-6) and linolenic (C18:3n-3) acids.

**Figure 2:**
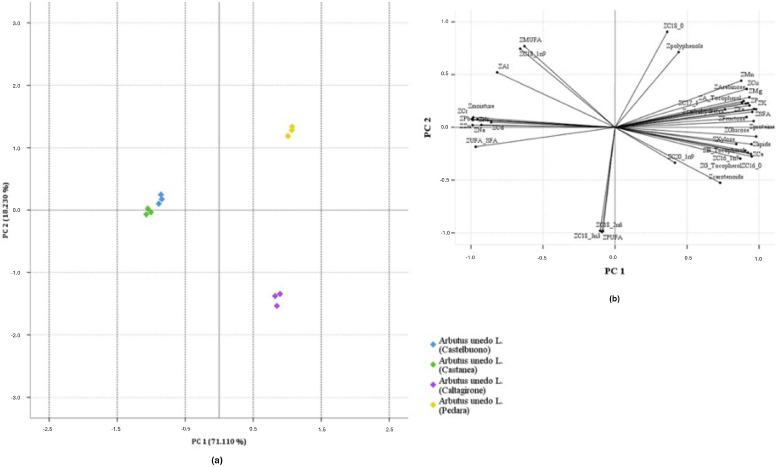
Bidimensional score (**a**) and loading (**b**) plots of PC2 vs. PC1 obtained by considering all experimental variables, excluding δ-tocopherol and maltose, during PCA analysis and showing the strawberry tree samples grouped according to the geographical origin.

Figure 2 shows the 2D scatterplots on the plane defined by PC1 and PC2 for the samples.

Four sample groups are clearly distinguishable on PC1, which explains 71.110% of the total variance, since the berries from the southeastern area of Sicily (i.e., sampling sites of Caltagirone and Pedara) resulted well differentiated from the strawberry trees harvested in the northeastern part of the island (i.e., sampling sites of Castelbuono and Castanea delle Furie). In addition, by considering the PC2, which explain a lower percentage of total variance (18.230%), fruits collected in Caltagirone are well separated from the strawberry trees sampled in Pedara; while there is little separation for the samples from the two areas of Castelbuono and Castanea.

Overall, the clustering of samples in the bidimensional space of PCs is well congruent with the discussion of the data made so far, since a strong influence of geographic origin on the nutritional profile of berries is evident. Specifically, the strawberry trees from Castelbuono and Castanea delle Furie had the most negative PC1 scores, and were grouped together because of their higher moisture degree, UFA/SFA ratio, along with greater concentrations of Pb, Zn and Na. The clustering of such fruit samples near each other was also influenced by shared lower concentrations of all the other inorganic elements, tocopherols, and soluble sugars. On the other hand, samples from Pedara and Caltagirone were characterized by the most positive and negative PC2 scores, respectively and hence, well separated on PC2 axis. Indeed, samples from Pedara were characterized by a higher polyphenol content and percentage of stearic acid (C18:0) than fruits from Caltagirone. On the other hand, strawberry trees from Caltagirone were well distinguished from berries collected in Pedara due to a higher content of linoleic (C18:2n-6) and linolenic (C18:3n-3) acids, and hence, total PUFA.

## 4. Conclusions

The present study intended not only to preserve and valorize the traditional and sustainable use of wild small fruits, but also to encourage research on neglected fruit species, such as the strawberry tree. In agreement with previous literature, this berry had a high dietary fiber content, significant levels of bioactives, such as tocopherols, polyphenols, and carotenoids, consistent amounts of minerals, and high levels of fructose, rather than glucose and sucrose, conferring it an intense sweet taste. These findings confirm that the strawberry tree not only is a good alternative to the wide range of common fruits for promoting health and wellness, but also an interesting raw material for developing food preservatives or, even, functional ingredients for functional foods and supplements. Additionally, novel evidence emerged from the study of Sicilian fruits. The linoleic acid/α-linoleic acid ratio found in the FA composition of these berries, could be relevant for discriminating between the Sicilian *A. unedo* fruit and strawberry trees from other regions. However, larger samples of Sicilian *A. unedo* should be further investigated, not only in terms of chemical composition of the fruits but also eco-physiological traits of the plant, to establish the linoleic acid/α-linoleic acid ratio as a reliable chemotaxonomic marker.

Additionally, berries grown in the proximity of Mount Etna were characterized by a higher mineral content and a greater abundance of bioactives than strawberry trees from other Sicilian areas, thus, advancing the hypothesis that fruits from volcanic areas may take advantage of a superior nutritional profile. Clearly, besides minerals and tocopherols, which have already been detailed in this study, further analyses on relevant secondary metabolites that may be affected by the exposure to volcanic ash (i.e., single polyphenols and carotenoids) should be conducted to confirm this hypothesis.

Overall, our data implemented with a suitable statistical analysis, confirmed that the geographical origin represented a relevant variable to consider in the study and valorization of the strawberry tree.

## Figures and Tables

**Table 1:** Detailed information on strawberry tree samples.

Sample Code	Geographical Origin	Geographical Coordinates	Sampling Date	No. of Replicates
S1	Castanea delle Furie (Messina, Italy)	38°15′46.2″ N 15°31′22.4″ E	November 2024	3
S2	Castelbuono (Palermo, Italy)	37°57′28.4″ N 14°05′20.3″ E		3
S3	Caltagirone (Catania, Italy)	37°14′14.2″ N 14°30′43.7″ E		3
S4	Pedara (Catania, Italy)	37°37′06.2″ N 15°03′42.4″ E		3

**Table 2:** Instrumental conditions of the ICP-MS analysis.

Parameter	Setting Value
Radio-frequency power	1500 W
Plasma gas flow rate (Ar)	14 L/min
Auxiliary gas flow rate (Ar)	0.8 L/min
Carrier gas flow rate (Ar)	1.1 L/min
Collision gas flow rate (He)	4.7 mL/min
Spray chamber temperature	2.7 °C
Injection volume	200 μL
Sample introduction flow rate	0.93 mL/min
Acquisition mode	Full scan mode
Integration time	0.5 or 0.1 s/point based on the analyte

**Table 3:** Proximate composition (%, fw) of Sicilian strawberry trees from different localities. Data are expressed as mean ± standard deviation of *n* = 3 samples per location, where every sample was analyzed in triplicate. S1: sample from Castelbuono (Palermo, Italy); S2: sample from Castanea delle Furie (Messina, Italy); S3: sample from Caltagirone (Catania, Italy); S4: sample from Pedara (Catania, Italy).

**Sample**	**Moisture**	**Carbohydrates**	**Protein**	**Lipids**	**Crude Fiber**	**Ash**
S1	56.27 ± 1.06 ^a^	18.00 ± 1.46 ^a^	1.63 ± 0.09 ^a^	1.65 ± 0.07 ^a^	9.88 ± 1.55 ^a^	1.13 ± 0.12 ^a^
S2	56.31 ± 0.59 ^a^	19.87 ± 0.63 ^a^	1.68 ± 0.04 ^a^	1.75 ± 0.12 ^a^	9.81 ± 2.40 ^a^	1.28 ± 0.10 ^a^
S3	46.82 ± 0.57 ^b^	22.53 ± 2.78 ^b^	2.50 ± 0.06 ^b^	2.43 ± 0.15 ^b^	12.84 ± 1.50 ^a^	2.00 ± 0.07 ^b^
S4	47.03 ± 0.90 ^b^	24.62 ± 2.62 ^b^	2.48 ± 0.07 ^b^	2.34 ± 0.12 ^b^	13.00 ± 2.37 ^a^	2.58 ± 0.11 ^c^

a–c: different superscript letters in the same column indicate significantly different values for a given parameter (*p* < 0.05 by post hoc Kruskal-Wallis test); same superscript letters in the same column indicate not significantly different values (*p* > 0.05 by post hoc Kruskal-Wallis test).

**Table 4:** Profile of sugars (g/100 g, fw) of Sicilian *A. unedo* berries from diverse sampling sites. Data are expressed as mean ± standard deviation of *n* = 3 samples per site, where every sample was analyzed in triplicate. S1: sample from Castelbuono (Palermo, Italy); S2: sample from Castanea delle Furie (Messina, Italy); S3: sample from Caltagirone (Catania, Italy); S4: sample from Pedara (Catania, Italy). <LOD: lower than the limit of detection of maltose (0.001 g/100 g).

Sample	Fructose	Glucose	Sucrose	Xylose	Arabinose	Maltose
S1	6.37 ± 0.56 ^a^	2.24 ± 0.30 ^a^	0.96 ± 0.08 ^a^	0.25 ± 0.09 ^a^	0.09 ± 0.02 ^a^	<LOD
S2	6.93 ± 0.44 ^a^	1.91 ± 0.24 ^a^	0.95 ± 0.11 ^a^	0.41 ± 0.10 ^a^	0.07 ± 0.02 ^a^	<LOD
S3	8.60 ± 0.39 ^b^	4.55 ± 0.72 ^b^	1.17 ± 0.17 ^a^	0.85 ± 0.10 ^b^	0.14 ± 0.04 ^b^	<LOD
S4	9.37 ± 0.42 ^b^	5.40 ± 0.81 ^b^	1.16 ± 0.06 ^a^	0.77 ± 0.08 ^b^	0.22 ± 0.03 ^c^	<LOD

a–c: different superscript letters in the same column indicate significantly different values for a given parameter (*p* < 0.05 by post hoc Kruskal-Wallis test); same superscript letters in the same column indicate not significantly different values (*p* > 0.05 by post hoc Kruskal-Wallis test).

**Table 5:** FA composition (%, fw) of Sicilian strawberry trees with different geographical origin. Data are expressed as mean ± standard deviation of *n* = 3 samples per location, where every sample was analyzed in triplicate. S1: sample from Castelbuono (Palermo, Italy); S2: sample from Castanea delle Furie (Messina, Italy); S3: sample from Caltagirone (Catania, Italy); S4: sample from Pedara (Catania, Italy). SFA: saturated fatty acids; MUFA: monounsaturated fatty acids; PUFA: polyunsaturated fatty acids; UFA: unsaturated fatty acids.

FA	S1	S2	S3	S4
C16:0	16.54 ± 0.30 ^a^	15.43 ± 0.20 ^b^	21.66 ± 0.12 ^c^	19.92 ± 0.18 ^d^
C16:1 n-7	0.32 ± 0.03 ^a^	0.23 ± 0.02 ^b^	0.64 ± 0.05 ^c^	0.52 ± 0.04 ^d^
C17:0	0.62 ± 0.04 ^a^	0.79 ± 0.04 ^a^	0.92 ± 0.18 ^b^	0.84 ± 0.06 ^a,b^
C17:1	1.37 ± 0.05 ^a^	1.14 ± 0.03 ^b^	1.56 ± 0.12 ^a^	1.78 ± 0.10 ^c^
C18:0	6.52 ± 0.18 ^a^	5.48 ± 0.37 ^b^	5.01 ± 0.10 ^b^	9.34 ± 0.24 ^c^
C18:1 n-9	18.27 ± 0.17 ^a^	19.20 ± 0.20 ^b^	7.37 ± 0.36 ^c^	15.86 ± 0.18 ^d^
C18:1 n-7	1.02 ± 0.07 ^a^	1.08 ± 0.03 ^a^	0.72 ± 0.05 ^b^	1.09 ± 0.04 ^a^
C18:2 n-6	33.30 ± 0.55 ^a^	34.32 ± 0.16 ^a^	37.35 ± 0.32 ^b^	30.58 ± 0.44 ^c^
C18:3 n-3	20.76 ± 0.25 ^a^	21.11 ± 0.03 ^a^	23.22 ± 0.72 ^b^	18.75 ± 0.45 ^c^
C20:0	1.04 ± 0.13 ^a^	0.84 ± 0.04 ^a^	0.97 ± 0.12 ^a^	0.84 ± 0.04 ^a^
C20:1 n-9	0.33 ± 0.07 ^a^	0.15 ± 0.02 ^b^	0.35 ± 0.04 ^a^	0.25 ± 0.04 ^a^
SFA	24.71 ± 0.15 ^a^	22.54 ± 0.27 ^b^	28.56 ± 0.10 ^c^	30.93 ± 0.45 ^d^
MUFA	21.32 ± 0.10 ^a^	21.80 ± 0.26 ^a^	10.65 ± 0.51 ^b^	19.50 ± 0.26 ^c^
PUFA	54.06 ± 0.80 ^a^	55.43 ± 0.16 ^a^	60.57 ± 0.70 ^b^	49.33 ± 0.20 ^c^
UFA/SFA	3.05 ± 0.05 ^a^	3.18 ± 0.24 ^b^	3.28 ± 0.24 ^c^	2.23 ± 0.05 ^d^
n-6/n-3	1.60 ± 0.01 ^a^	1.63 ± 0.01 ^a^	1.61 ± 0.05 ^a^	1.63 ± 0.06 ^a^

a–d: different superscript letters in the same row indicate significantly different values for a given parameter (*p* < 0.05 by post hoc Kruskal-Wallis test); same superscript letters in the same row indicate not significantly different values (*p* > 0.05 by post hoc Kruskal-Wallis test).

**Table 6:** Content of tocopherols (mg/kg, fw) in Sicilian strawberry trees from different localities. Data are expressed as mean ± standard deviation of *n* = 3 samples per location, where every sample was analyzed in triplicate. S1: sample from Castelbuono (Palermo, Italy); S2: sample from Castanea delle Furie (Messina, Italy); S3: sample from Caltagirone (Catania, Italy); S4: sample from Pedara (Catania, Italy). <LOD: lower than the limit of detection (0.01 mg/kg).

Sample	α-Tocopherol	β-Tocopherol	γ-Tocopherol	δ-Tocopherol	Total
S1	6.42 ± 0.32 ^a^	0.08 ± 0.01 ^a^	2.05 ± 0.18 ^a^	<LOD	8.55 ± 0.50 ^a^
S2	6.23 ± 0.34 ^a^	0.12 ± 0.02 ^a^	2.29 ± 0.17 ^a^	<LOD	8.65 ± 0.36 ^a^
S3	7.64 ± 0.33 ^b^	0.35 ± 0.06 ^b^	3.20 ± 0.16 ^b^	<LOD	11.19 ± 0.32 ^b^
S4	8.58 ± 0.39 ^c^	0.28 ± 0.08 ^a^	2.87 ± 0.13 ^b^	<LOD	11.73 ± 0.33 ^b^

a–c: different superscript letters in the same column indicate significantly different values for a given parameter (*p* < 0.05 by post hoc Kruskal-Wallis test); same superscript letters in the same column indicate not significantly different values (*p* > 0.05 by post hoc Kruskal-Wallis test).

**Table 7:** Total carotenoids (mg BCE/kg, fw) and polyphenols (mg GAE/100 g, fw) of Sicilian *A. unedo* fruits from different sampling sites. Data are expressed as mean ± standard deviation of *n* = 3 samples per site, where every sample was analyzed in triplicate. S1: sample from Castelbuono (Palermo, Italy); S2: sample from Castanea delle Furie (Messina, Italy); S3: sample from Caltagirone (Catania, Italy); S4: sample from Pedara (Catania, Italy).

Sample	Total Carotenoids	Total Polyphenols
S1	11.73 ± 0.23 ^a^	3324.27 ± 119.85 ^a,b^
S2	9.60 ± 0.50 ^b^	3027.61 ± 138.90 ^a^
S3	15.45 ± 0.17 ^c^	3100.17 ± 140.26 ^a^
S4	12.11 ± 0.17 ^a^	3666.17 ± 231.04 ^b^

a–c: different superscript letters in the same column indicate significantly different values for a given parameter (*p* < 0.05 by post hoc Kruskal-Wallis test); same superscript letters in the same column indicate not significantly different values (*p* > 0.05 by post hoc Kruskal-Wallis test).

**Table 8:** Elemental profile (mg/kg, fw) of Sicilian *A. unedo* fruits with different geographical origin. Data are expressed as mean ± standard deviation of *n* = 3 samples per location, where every sample was analyzed in triplicate. S1: sample from Castelbuono (Palermo, Italy); S2: sample from Castanea delle Furie (Messina, Italy); S3: sample from Caltagirone (Catania, Italy); S4: sample from Pedara (Catania, Italy). <LOQ: lower than the limit of quantification of Pb (0.007 mg/kg).

Element	S1	S2	S3	S4
Na	224.01 ± 5.84 ^a^	234.48 ± 15.49 ^a^	194.92 ± 4.37 ^b^	192.97 ± 5.28 ^b^
Ca	948.61 ± 20.99 ^a^	938.87 ± 25.65 ^a^	1286.03 ± 45.90 ^b^	1196.50 ± 74.36 ^b^
Mg	113.11 ± 7.54 ^a^	114.31 ± 6.15 ^a^	148.68 ± 5.77 ^b^	182.03 ± 7.73 ^c^
K	2517.74 ± 45.24 ^a^	2435.99 ± 42.48 ^a^	3425.87 ± 60.28 ^b^	3940.53 ± 184.06 ^c^
P	736.96 ± 6.71 ^a^	733.84 ± 9.81 ^a^	900.33 ± 56.85 ^b^	951.98 ± 51.20 ^b^
Zn	7.03 ± 0.14 ^a^	7.15 ± 0.15 ^a^	5.30 ± 0.18 ^b^	5.16 ± 0.09 ^b^
Fe	6.11 ± 0.13 ^a^	5.48 ± 0.34 ^a^	7.74 ± 0.13 ^b^	9.27 ± 1.43 ^b^
Cu	0.83 ± 0.06 ^a^	0.88 ± 0.05 ^a^	1.47 ± 0.10 ^b^	2.64 ± 0.10 ^c^
Mn	0.60 ± 0.06 ^a^	0.61 ± 0.09 ^a^	1.03 ± 0.12 ^b^	2.00 ± 0.08 ^c^
Mo	0.031 ± 0.010 ^a^	0.036 ± 0.008 ^a^	0.017 ± 0.009 ^a^	0.016 ± 0.006 ^a^
Cr	1.71 ± 0.07 ^a^	1.72 ± 0.14 ^a^	0.76 ± 0.06 ^b^	0.75 ± 0.03 ^b^
Ni	0.033 ± 0.011 ^a^	0.032 ± 0.013 ^a^	0.010 ± 0.009 ^a^	0.011 ± 0.010 ^a^
Al	4.14 ± 0.08 ^a^	4.16 ± 0.11 ^a^	3.35 ± 0.09 ^b^	3.77 ± 0.15 ^c^
Cd	0.022 ± 0.006 ^a^	0.020 ± 0.005 ^a^	0.015 ± 0.003 ^a^	0.012 ± 0.010 ^a^
Pb	0.012 ± 0.004 ^a^	0.014 ± 0.007 ^a^	<LOD	<LOD
As	0.013 ± 0.002 ^a^	0.015 ± 0.005 ^a^	0.013 ± 0.003 ^a^	0.011 ± 0.002 ^a^

a–c: different superscript letters in the same row indicate significantly different values for a given parameter (*p* < 0.05 by post hoc Kruskal-Wallis test); same superscript letters in the same row indicate not significantly different values (*p* > 0.05 by post hoc Kruskal-Wallis test).

## Data Availability

Dataset available on request from the authors.
